# Switched Aβ43 generation in familial Alzheimer’s disease with presenilin 1 mutation

**DOI:** 10.1038/s41398-021-01684-1

**Published:** 2021-11-03

**Authors:** Nobuto Kakuda, Mako Takami, Masayasu Okochi, Kensaku Kasuga, Yasuo Ihara, Takeshi Ikeuchi

**Affiliations:** 1 Department of Neuropathology, Faculty of Life and Medical Sciences, Doshisha University, Kyoto, 610-0394 Japan; 2 Center for Research in Neurodegenerative Diseases, Doshisha University, Kyoto, 610-0394 Japan; 3 Neuropsychiatry and Neurochemistry, Department of Integrated Medicine, Division of Internal Medicine, Osaka University Graduate School of Medicine, Osaka, 565-0871 Japan; 4 Department of Molecular Genetics, Bioresource Science Branch, Center for Bioresources, Niigata University, Niigata, 951-8585 Japan

**Keywords:** Clinical genetics, Molecular neuroscience

## Abstract

Presenilin (PS) with a genetic mutation generates abundant β-amyloid protein (Aβ) 43. Senile plaques are formed by Aβ43 in the cerebral parenchyma together with Aβ42 at middle ages. These brains cause the early onset of Alzheimer’s disease (AD), which is known as familial Alzheimer’s disease (FAD). Based on the stepwise processing model of Aβ generation by γ-secretase, we reassessed the levels of Aβs in the cerebrospinal fluid (CSF) of FAD participants. While low levels of Aβ38, Aβ40, and Aβ42 were generated in the CSF of FAD participants, the levels of Aβ43 were unchanged in some of them compared with other participants. We sought to investigate why the level of Aβ43 was unchanged in FAD participants. These characteristics of Aβ generation were observed in the γ-secretase assay in vitro using cells, which express FAD mutations in PS1. Aβ38 and Aβ40 generation from their precursors, Aβ42 and Aβ43, was decreased in PS1 mutants compared with wild-type (WT) PS1, as observed in the CSF. Both the ratios of Aβ38/Aβ42 and Aβ40/Aβ43 in PS1 mutants were lower than those in the WT. However, the ratio of Aβ43/amyloid precursor protein intracellular domain (AICD) increased in the PS1 mutants in an onset age dependency, while other Aβ/AICD ratios were decreased or unchanged. Importantly, liquid chromatography–mass spectrometry found that the generation of Aβ43 was stimulated from Aβ48 in PS1 mutants. This result indicates that PS1 mutants switched the Aβ43 generating line, which reflects the level of Aβ43 in the CSF and forming senile plaques.

## Introduction

Familial Alzheimer’s disease (FAD) patients are less prevalent (approximately 1%) than those with sporadic Alzheimer’s disease (AD) [1, 2]. More than 200 genetic mutations have been found in presenilin (PS) 1 and 50 mutations in PS2, which are the catalytic core of the γ-secretase complex [3–5], and >60 mutations in amyloid precursor protein (APP) have been discovered [6]. APP is cleaved sequentially by β- and γ-secretase to generate amyloid β-protein (Aβ) proteins [4]. FAD mutations in PS and APP increase the ratio of Aβ42/Aβ40 compared with the ratio in wild-type (WT) [7–10]. Senile plaques, a neuropathological hallmark of AD, are composed largely of Aβ42 [11]. Thus, Aβ42 has been considered the earliest species deposited in the parenchyma and the real culprit for the development of sporadic AD. In addition, Aβ43, a longer but much less prevalent Aβ, has been found in the senile plaques of the brains of those affected by AD and Down syndrome [12–14]. Nishimura and colleagues reported that random mutagenesis generated a PS1 R278I mutation that results exclusively in the production of Aβ43 [15], and Saido and colleagues reported that mutant PS1 transgenic mice (R278I/APP^sw^) had elevated levels of Aβ43 and earlier formation of senile plaques in their brains [16]. This particular mutation was identified in a patient who presented with language disturbance [17]. Recently, Aβ43 generation and deposition were found to be increased in the brains of those carrying FAD mutations in PS1 [13, 14]. These studies raise the possibility that Aβ43 plays a pivotal role in the development of FAD.

γ-Secretase generates each Aβ under two pathways. At first, Aβ49 and Aβ48 are cleaved from APP. Aβ49 is successively cleaved, mostly to Aβ40, via Aβ46 and Aβ43, while Aβ48 is similarly cleaved to Aβ38 via Aβ45 and Aβ42. Of note, the most abundant species, Aβ40, is derived not from Aβ42 but from Aβ43. Moreover, Aβ38 is derived mainly from Aβ42 and Aβ43 [18–20]. These sequential Aβ generation mechanisms have been referred to as stepwise processing [18–20].

We previously reported that (i) the levels of Aβ42 and Aβ43 are proportional, and the levels of Aβ38 and Aβ40 are also proportional in the cerebrospinal fluid (CSF) of sporadic AD participants, mild cognitive impairment (MCI) participants, and cognitively normal controls (NCs). The levels of Aβ concentration in the CSF might reflect lipid raft-associated γ-secretase activity from brain cortices [21, 22]; (ii) there is a correlation between the levels of deposited Aβ42 and Aβ43 in the cerebral parenchyma in the NCs, MCI, and sporadic AD brains [23]; and (iii) lipid raft-nonassociated γ-secretase activity in the AD brains increases the generation of Aβ42 and Aβ43 from brain cortices, and these released Aβs into the extracellular space probably form senile plaques [23]. Thus, not only Aβ in the CSF but also its deposition in the brain parenchyma depends on γ-secretase activities.

Based on these characteristics of γ-secretase in sporadic AD, we reassessed the mechanism of Aβ generation and γ-secretase activity in PS1 mutants using human CSF and in vitro γ-secretase assays. CSF samples from FAD participants showed low levels of Aβ38, Aβ40, Aβ42, and Aβ43 but not some Aβ43. Surprisingly, although lipid raft-associated γ-secretase activity, which was prepared from mutant PS1 stably expressing cells, showed decreased Aβ38, Aβ40, and some Aβ42 generation in the in vitro γ-secretase assay, the level of Aβ43 was the same as the level of WT PS1. This γ-secretase activity was distinctly different from the alteration of γ-secretase activity found in those with sporadic AD [21]. Liquid chromatography–mass spectrometry (LC-MS/MS) revealed that this altered Aβ43 generation was not only from Aβ46 but also from Aβ48 in the PS1 mutants, despite different stepwise processing pathways. The extent of switching Aβ43 generation might reflect FAD onset.

## Materials and methods

### Participants

The previously measured CSF samples of participants with sporadic AD, MCI, and cognitively NCs were described previously [21]. Briefly, we included 24 participants with AD (mild-to-moderate AD; 50–86 years old), 19 participants with MCI (57–82 years old), and 21 cognitively NC participants (61–89 years old). The CSF samples from 5 participants with (symptomatic) FAD (mutant PS1; T116N, L173F, G209R, L286V, and L381V) were from Niigata University Hospital, and 1 patient with FAD (mutant PS1; L85P) was from Osaka University Hospital.

### CSF analysis

CSF (10–15 mL) was collected in a polypropylene or polystyrene tube and gently inverted. After brief centrifugation, aliquots of CSF collected were transferred to polypropylene tubes (0.25–0.5 mL), which were kept at −80 °C until use. The CSF concentrations of Aβ38, Aβ40, Aβ42, and Aβ43 were quantified using commercially available enzyme-linked immunosorbent assay kits (cat NOs. 27717, 27718, 27712, and 27710, respectively, IBL, Gunma, Japan).

### Cell culture

A previously reported cell culture method was employed [24]. Briefly, WT or mutant PS1 stably expressing Chinese hamster ovary (CHO) or human embryo kidney (HEK) 293 cells were cultured in Dulbecco’s modified Eagle’s medium (Sigma, St. Louis, MO, USA) containing 10% fetal bovine serum (Invitrogen, Carlsbad, CA, USA) and penicillin/streptomycin (Invitrogen). In these PS1-expressing cell lines (Table S1), displacement of endogenous PS1 was confirmed by western blotting [25, 26].

### Quantification of raft-associated γ-secretase activity

A previously reported assay method was employed with some modifications [21, 27]. Briefly, raft fractions were collected from each membrane fraction of the cell. The protein concentration of each raft fraction was adjusted to 100 μg/mL and then incubated with 500 nM FLAG-tagged β carboxyl-terminal fragment (βCTF) (C99-FLAG) for 1 h at 37 °C. The proteins in the samples were separated using sodium dodecyl sulfate–polyacrylamide gel electrophoresis and subjected to quantitative western blotting using specific antibodies, 3B1 for Aβ38, BA27 for Aβ40, 44A3 for Aβ42, anti-Aβ43 polyclonal for Aβ43 (IBL, Gunma Japan), and amyloid intracellular domain (AICD) for UT-421 (kindly gifted by Dr. T. Suzuki, Hokkaido University).

### LC-MS/MS quantification of released peptides

The expected peptides were quantified using LC-MS/MS as previously described [18–20]. A Quattro Premier XE tandem quadrupole mass spectrometer in tandem with ultra-high-performance liquid chromatography (Waters system equipped with an Acquity UPLC HSS T3 column, 1.8 μm, 2.0 × 150 mm) was used to identify and quantify the released oligopeptides.

### Statistical analysis

All statistical analyses were conducted using GraphPad Prism version 8. Data transformation was required to achieve normal distributions in the CSF analysis. Data analyses were performed after logarithmic transformation of the data for Aβ38, Aβ40, Aβ42, and Aβ43 and compared with our previous results. In the case of Aβ generation in the in vitro assay, Pearson’s correlation coefficients were calculated to indicate the strength of the relationship between two variables.

## Results

### Altered Aβ43 generation in the PS mutations

**Fig. 1:**
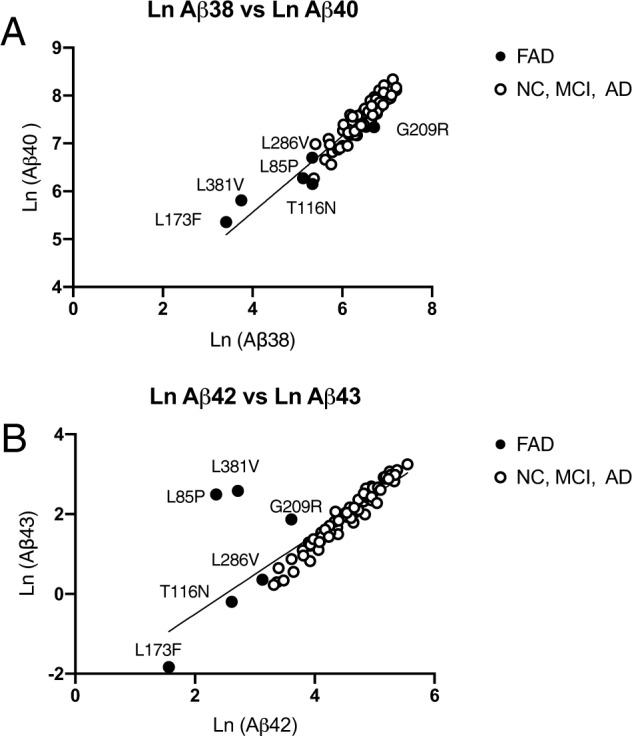
The relationship between the level of each Aβ in the CSF from FAD participants and the levels in cognitively normal controls, MCI participants, and AD participants. **A** The levels of Ln Aβ40 were proportional to Ln Aβ38 in all subjects. **B** The levels of Ln Aβ43 were proportional to Ln Aβ42, except for the PS1 mutation in L85P, G209R, and L381V.

Honorable previous studies reported that many PS1 mutants have lower and higher γ-secretase activity to generate Aβs than WT PS1 [28–31]. These altered γ-secretase activities are probably reflected in the level of Aβ found in CSF. The Aβ levels in the CSF of participants with FAD were compared with those in cognitively NC participants, MCI participants, and AD participants, as found in our previous study [21] (Fig. 1). The Aβ levels in FAD participants were lower than those we previously reported [21], except for one case of FAD (G209R) in the Ln Aβ38 versus Ln Aβ40 plot and three cases of FAD (L85P, G209R, and L381V) in the Ln Aβ42 versus Ln Aβ43 plots (Fig. 1). Interestingly, a clear proportion of Aβ38 and Aβ40 was observed in the CSF from FAD participants compared with other participants (Fig. 1A; Ln (Aβ40) = 0.7105 × (Ln Aβ38) + 2.78, *R* = 0.9536). These Aβs are the set of major final products in the stepwise processing of Aβ. When Aβ38 generation from Aβ42 decreased in the CSF of FAD participants, Aβ40 generation from Aβ43 also decreased, as found in the CSF of other participants. This proportional plot indicates that both γ-secretase cleavages, from Aβ43 to Aβ40 and from Aβ42 to Aβ38, occurred simultaneously in FAD brains, as found in other participants. However, the plots of Aβ42 and Aβ43 in the FAD participants varied within those of other participants. In these plots, the levels of the FAD participants (L85P, G209R, and L381V) digressed far from the regression line (Fig. 1B; Ln (Aβ43) = 0.9963 × (Ln Aβ42) − 2.497, *R* = 0.8333). The onset age of these mutations was 27 years for L85P, 49.6 years for G209R, and 29 years for L381V. These results indicate that the levels of Aβ38, Aβ40, and Aβ42 decreased in the CSF of FAD participants, while Aβ43 might be defective by those mutations (Fig. 1B).

### The γ-secretase activity of FAD mutation in the PS1

**Fig. 2:**
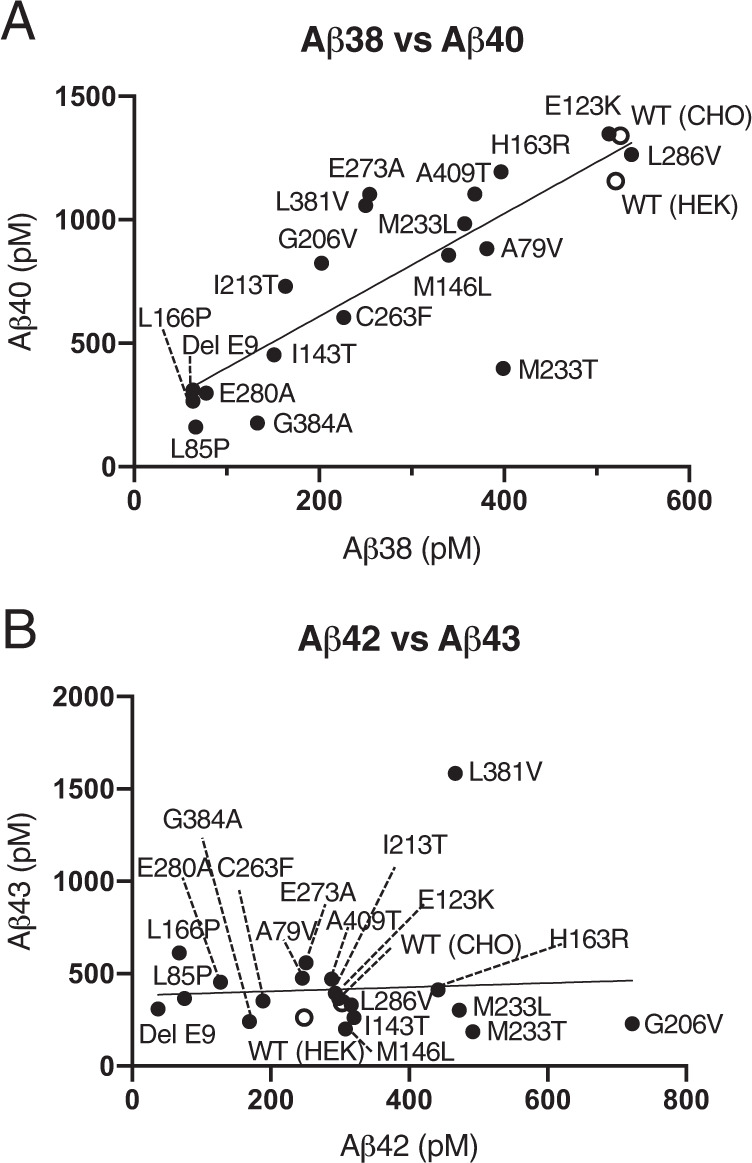
Levels of each Aβ were generated based on a γ-secretase assay in vitro. **A** The levels of Aβ40 were proportional to the levels of Aβ38 in cells expressing WT or mutant PS1. **B** The levels of Aβ43 were unchanged in PS1 mutants. Information about all PS1 mutations and the levels of Aβ by in vitro assay is indicated in Table S1. The open circles indicate WT PS1, and the closed circles indicate mutant PS1. (*n* = 3).

To provide further insight into the effects of PS1 mutation on the stepwise Aβ processing mechanism, we directly measured γ-secretase activities using CHO and HEK293 cells. These cells expressed WT or FAD mutant PS1, which forms γ-secretase [24, 28]. The lipid raft fractions, which contain γ-secretase, were isolated from these PS1-expressing cells and are referred to as raft-associated γ-secretase. In our previous study, lipid raft-associated γ-secretase from human brain cortices generated a ratio of Aβs similar to Aβs in CSF [21]. These lipid raft fractions were incubated with their substrate, C99-FLAG, to assess each mutant PS1 γ-secretase activity. This in vitro assay showed proportional plots between Aβ38 and Aβ40, as observed in the case of CSF (Fig. 2A; Aβ40 = 2.082 × Aβ38 + 192.5, *R* = 0.8412). The highest levels of Aβ40 and Aβ38 were in the WT PS1 from both CHO and HEK293 cells (Fig. 2A). These results are consistent with the previous finding by Van Broeckhoven and colleagues [32]. In contrast, both increased and decreased levels of Aβ42 were observed in the PS1 mutants compared with those of WT PS1 (Fig. 2B; Aβ43 = 0.1119 × Aβ42 + 382.2, *R* = 0.06276). Although Aβ42 decreased in some PS1 mutants, the level of Aβ43 was unchanged, as observed in the CSF of three early-onset FAD mutants (Figs. 1B and 2B).

**Fig. 3:**
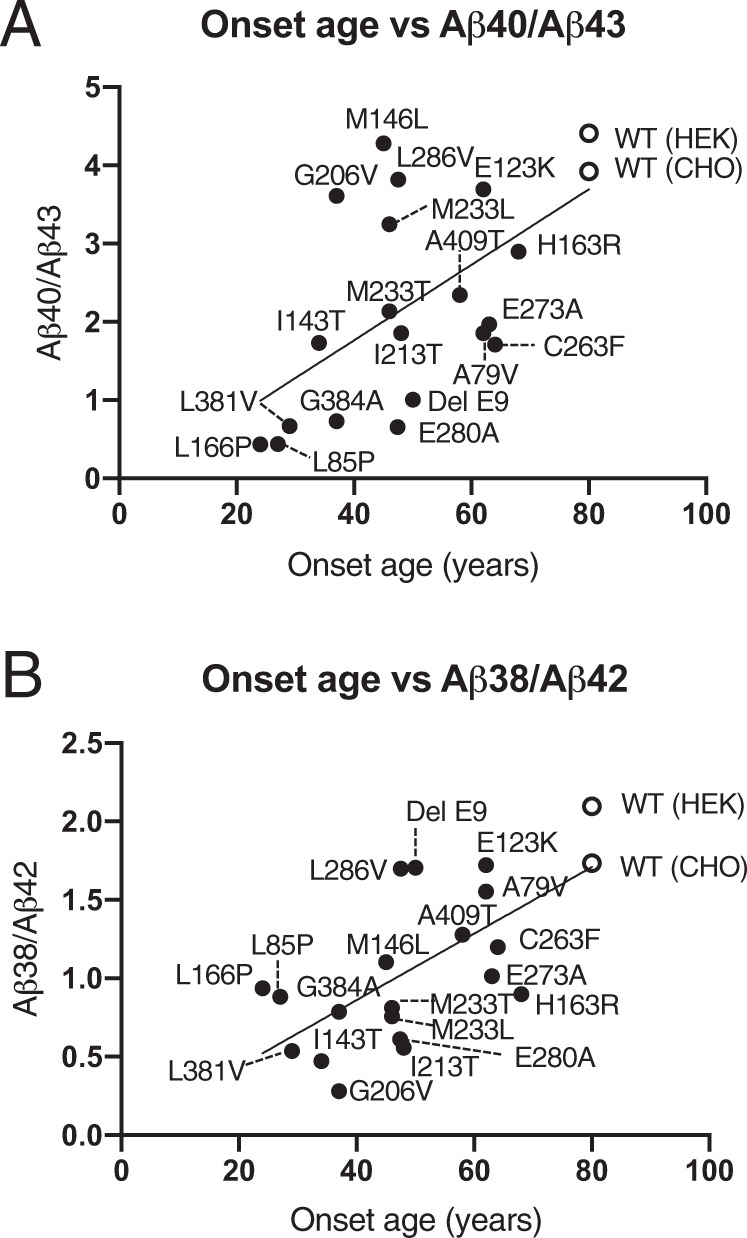
Onset age versus the ratio of Aβ for each FAD mutation by γ-secretase assay in vitro. **A**, **B** The ratio of Aβ40/Aβ43 or Aβ38/Aβ42 versus onset age is on the regression line. These plots suggest onset age dependency. The open circles indicate WT PS1, and the closed circles indicate FAD mutant PS1. (*n* = 3).

Next, we compared the ratios of Aβ40/Aβ43 and Aβ38/Aβ42, which are Aβ product/precursor sets, with each AD onset age. Although Aβ43 generation was unchanged in the PS1 mutants (Fig. 2B), these Aβ40/Aβ43 versus onset age plots were weakly proportional, as previously reported [16] (Fig. 3A; Aβ40/Aβ43 = 0.04822 × (onset age) − 0.1641, *R* = 0.5783). These reductions in Aβ40 occurred in an onset age dependency, as previously reported [16, 32]. Another Aβ generation pathway Aβ38/Aβ42 versus onset age was also proportional and onset age dependent (Fig. 3B; Aβ38/Aβ42 = 0.02120 × (onset age) + 0.01322, *R* = 0.6751). These results indicated that FAD mutations of the early-onset ages have inhibitory effects on both γ-secretase-mediated cleavage pathways, from Aβ43 to Aβ40 and from Aβ42 to Aβ38.

### Aβ43 generation increased FAD onset age dependency

**Fig. 4:**
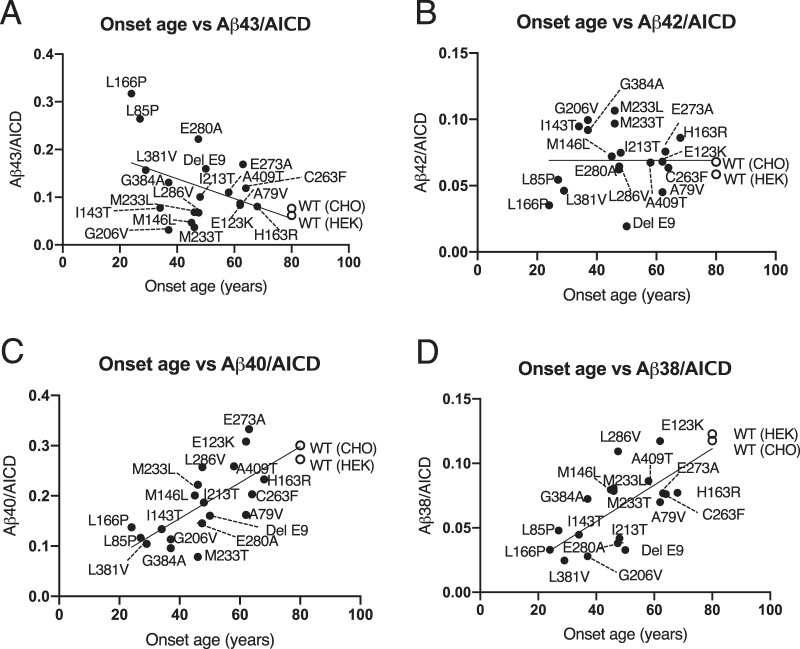
Onset age versus the ratios of Aβ/AICD for each FAD mutation. **A** The ratio of Aβ43/AICD increased with age-dependent onset. **B** The ratio of Aβ42/AICD was almost unchanged at all onset ages. **C** The ratio of Aβ40/AICD and **D** Aβ38/AICD decreased with onset age dependency. The open circles indicate WT PS1, and the closed circles indicate FAD mutant PS1. (*n* = 3).

In the Aβ-generating mechanism, γ-secretase cleaves βCTF to generate Aβ48 and Aβ49 at first, which is known as ε-cleavage, and then those counterparts of the AICD are released [24, 33, 34]. In the case of PS1 mutants, some Aβ species decreased, but AICD generation was almost unchanged in the in vitro assay (Fig. S1). Previously, we showed that the level of total Aβ was equal to the level of total AICD in the in vitro γ-secretase assay [24]. Thus, the levels of AICD showed the level of total Aβ in this assay. In the present study, the ratio of Aβ/AICD was used to compare with the onset age of FAD. Although the ratio of Aβ43/AICD showed a correlation in PS1 mutant onset age dependency, other ratios of Aβ/AICD were decreased or unchanged (Fig. 4A; Aβ43/AICD = −0.00237 × (onset age) + 0.2342, *R* = −0.4466). The ratio of Aβ42/AICD was found to be almost constant (Fig. 4B; Aβ42/AICD = 0.0001172 × (onset age) + 0.06415, *R* = −0.001039). These findings demonstrate that the levels of Aβ42 generation depend on the value of AICD generation. However, the level of Aβ43 generation might be the independent manner of PS1 mutants. Other ratios, Aβ38/AICD and Aβ40/AICD, were reduced in an onset age dependency (Fig. 4C; Aβ40/AICD = 0.003806 × (onset age) + 0.002352, *R* = 0.7560, Fig. 4D; Aβ38/AICD = 0.001229 × (onset age) + 0.006055, *R* = 0.7350). Decreasing the values of Aβ38 and Aβ40 means increasing those of Aβ42 and Aβ43 (Fig. 3). Both γ-secretase cleavages, from Aβ43 to Aβ40 and from Aβ42 to Aβ38, seem to be tightly regulated by PS1 mutations (Fig. 4). Thus, the Aβ42/Aβ40 ratio would be increased in FAD mutants compared with WT PS1 [7–10]. However, the generation of Aβ43 might have another generation pathway by PS1 mutations.

### Aβ43 arises directly from Aβ48 in the FAD mutants

**Fig. 5:**
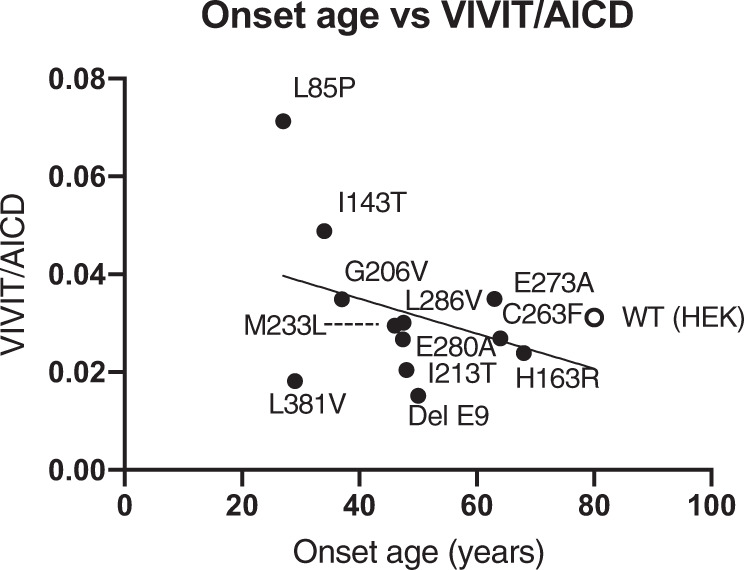
FAD onset age versus the ratio of VIVIT/AICD. VIVIT was released from Aβ48 to generate Aβ43 by γ-secretase cleavage. The VIVIT/AICD ratio increased with onset age dependency. WT and mutant PS1 (L85P, I143T, H163R, L166P, G206V, I213T, M233T, C263F, E273A, E280A, L286V, G384A, and del E9) were used in this assay. The open circle indicates WT PS1, and the closed circles indicate FAD mutant PS1. (*n* = 3).

A most important issue: “Where does Aβ43 come from in the case of PS1 mutants?” Previously, we reported that γ-secretase generates Aβ42 from Aβ48 via Aβ45 and Aβ43 from Aβ49 via Aβ46 stepwise processing pathway in the case of WT PS1 [18]. Aβ43 is derived mainly from Aβ46 but also a minority from Aβ47 and Aβ48 by raft-associated γ-secretase in rat brain [20]. Thus, we measured released peptides using LC-MS/MS to provide further insight into Aβ43 generating mechanism by an in vitro lipid raft-associated γ-secretase assay with each PS1 mutant. First, we compared each released peptide (Fig. S2A). In stepwise Aβ processing, ITL was generated from Aβ49 to Aβ46, and VIV was generated from Aβ46 to Aβ43. These peptides have a clear correlation even if a mutation exists in PS1 (Fig. S2B; ITL = 1.004 × VIV − 0.1709, *R* = 0.9997). On the other hand, VIT and TVI were generated from Aβ48 to Aβ45 and from Aβ45 to Aβ42 (Fig. S2A). These peptides also showed a correlation (Fig. S2C; VIT = 1.033 × TVI + 176.5, *R* = 0.9790). These clear correlations indicated that the levels of Aβ42 and Aβ43 generation depend on the levels of Aβ48 and Aβ49 generated by FAD mutation in PS1 (Fig. S2A, B). However, there seems to be a contradiction regarding Aβ43 generation, as shown in Fig. 4A. Therefore, we measured the ratios of each generated peptide/AICD compared with onset age. γ-Secretase releases VVIA and IAT to generate Aβ38 from Aβ42 and Aβ40 from Aβ43, respectively. The ratios of VVIA/AICD and IAT/AICD showed a similar pattern, as shown in Fig. 4C, D (Fig. S3). The ratios of ITL (from Aβ49 to Aβ46)/AICD, VIV (from Aβ46 to Aβ43)/AICD, VIT (from Aβ48 to Aβ45)/AICD, and TVI (from Aβ45 to Aβ42)/AICD decreased or remained unchanged depending on the onset age, as shown in Fig. 4B–D (Fig. S4). Importantly, the ratio of VIVIT/AICD increased onset age dependency, as observed in Fig. 4A of the ratio of Aβ43/AICD (Fig. 5; VIVIT/AICD = −0.0005321 × (onset age) + 0.05662, *R* = −0.3861). VIVIT was released from Aβ48 to Aβ43 by γ-secretase (Fig. S2A). These findings indicated that the PS1 mutation would be altered to generate Aβ43 from Aβ48 by the switching generation mechanism because Aβ48 generates Aβ42 via Aβ45 under WT PS1. However, Aβ40 decreased onset age dependency, although Aβ43 increased in PS1 mutants. Thus, these γ-secretase alterations reflect the level of Aβ43 in the CSF and Aβ43 might form senile plaques, including Aβ42, in the brains of FAD patients [14].

## Discussion

Here we assume that (i) the levels of Aβ38 and Aβ40 were on the regression line in the CSF; (ii) some levels of Aβ43 from FAD participants, but not those of Aβ42, diverged from the regression line; (iii) the in vitro γ-secretase assay followed these Aβ generations by PS1 mutations, as seen in the CSF; (iv) the levels of Aβ43/AICD reflected the onset age of FAD; and (v) Aβ43 was generated not only from Aβ46 but also from Aβ48 in the PS1 mutants. With these assumptions, the most important issue for the mechanism of Aβ43 generation in the PS1 mutant is that the Aβ43 generation line was switched from Aβ48. This is in contrast with the mechanism of γ-secretase activity in sporadic AD [21, 22].

In the present study, we quantified the concentrations of four Aβs, Aβ38, Aβ40, Aβ42, and Aβ43, in the CSF from FAD participants for comparison with our previous study [21]. Even if the patient had FAD mutations in PS1, Aβ38 and Aβ40 were clearly on the regression line, although absolute levels were lower than in others (Fig. 1A). Previous studies found that absolute Aβ40 generation decreased in FAD mutants in an onset age dependency [16, 28]. In our study, ε-cleavage by mutant PS1 was unchanged compared with WT PS1 because the level of AICD was unchanged (Fig. S1). Thus, we compared the Aβ38/AICD and Aβ40/AICD ratios (Fig. 4C, D). These ratios, for both Aβ38 and Aβ40 generation, were decreased in an onset age dependency. These results indicate that FAD mutants especially affect the decrease in Aβ38 and Aβ40 generation.

Next, the CSF concentrations of both Aβ42 and Aβ43 were proportional except in some early-onset ages of PS1 mutants (Fig. 1B). In the in vitro assays, the levels of Aβ42 decreased, but some Aβ43 did not. Similar plots were obtained in the PS1 mutant cells in the in vitro assay (Fig. 2B). In the case of a human, who has a mutation in the PS, as a heterozygote, but in the case of cells, it exists as a homozygote. This genetic difference would have appeared in the level of Aβ43 in the in vitro assay. As a result, the ratios of Aβ43/AICD, but not Aβ42/AICD, increased in an onset age-dependent manner (Fig. 4A, B). This finding indicates that the absolute Aβ42 level is determined by ε-cleavage efficacy in PS1 mutants. Importantly, a high concentration of Aβ43 might accelerate the formation of senile plaques in the brains, including Aβ42. Aβ43 induces aggregation more than Aβ42 by 1.5–2 times [16]. First, Aβ43 might need Aβ42 to form senile plaques in the cerebellum parenchyma because immunostaining revealed that both Aβ42 and Aβ43 exist in the same senile plaque [23]. Second, decreasing Aβ40 would increase Aβ42 aggregation in the brain. BRI-Aβ42/Tg2576 bitransgenic mice exhibited increased Aβ deposition compared with Tg2576 mice, but BRI-Aβ40/Tg2576 bitransgenic mice did not [35, 36]. Thus, the presence of Aβ40 prevents Aβ aggregation in the brain. Increasing Aβ43 and decreasing Aβ40 are the most important issues in PS1 mutation. The imbalance in the generation of these Aβs probably determines the onset age of FAD. We do not know why cross-talk generation of Aβ43 occurred in PS1 mutants. However, we previously found that the ratio of AICD49–99/50–99 was increased in PS and APP mutants compared with WT [34]. In addition, when T714I and V717F mutations exist in βCTF, γ-secretase generates mainly AICD49–99 in an in vitro assay [24]. In these mutants, Aβ48 might probably be a priority generation species. There is a contradiction because Aβ42 is generated from Aβ48 in the stepwise processing WT PS1 and APP. However, Aβ43 is generated by the V717F mutation, as seen in WT βCTF, although AICD50–99 could not detect, which is a counterpart of Aβ49 [24]. If FAD mutations are in γ-secretase or APP, the generation of Aβ48 would increase compared with that of WT. This alteration enhances Aβ43 generation by cross-talk of stepwise processing (Figs. 5 and S2A). Thus, the level of Aβ43 might be reflected in the CSF of FAD participants and those of onset age.

## Supplementary information

Table S1

Fig. S1

Fig. S2

Fig. S3

Fig. S4

Supplementary legends
