# Supplementary material for: Switched Aβ43 generation in familial Alzheimer’s disease with presenilin 1 mutation
Source: Transl Psychiatry. 2021 Nov 3;11:558. doi: 10.1038/s41398-021-01684-1 (PMC8564532; doi:10.1038/s41398-021-01684-1)
Supplement: Supplementary file 1 — Table S1 [file 41398_2021_1684_MOESM1_ESM.pptx]

## Slide 1
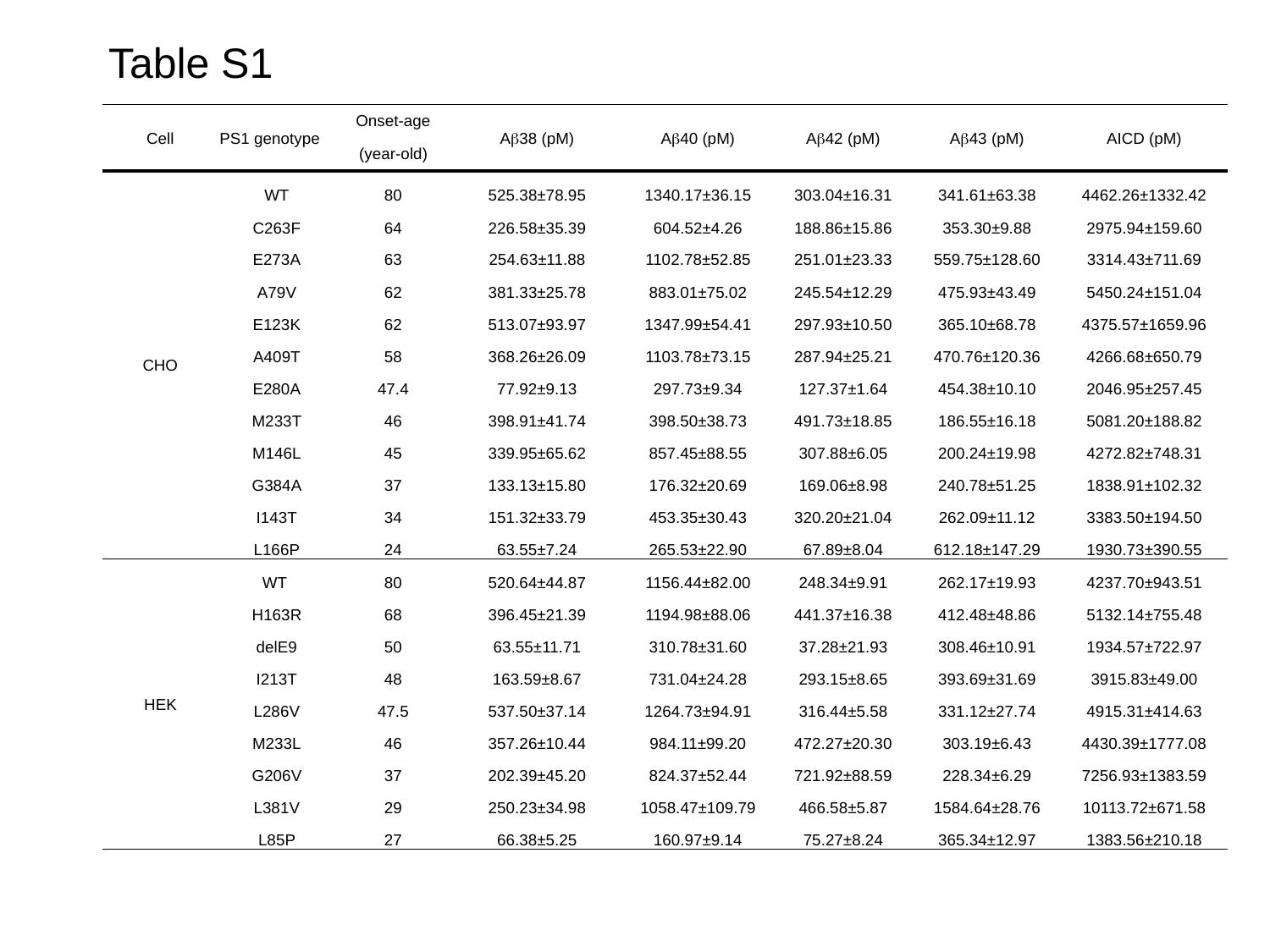

Table S1
| Cell | PS1 genotype | Onset-age | Ab38 (pM) | Ab40 (pM) | Ab42 (pM) | Ab43 (pM) | AICD (pM) |
| --- | --- | --- | --- | --- | --- | --- | --- |
| | | (year-old) | | | | | |
| CHO | WT | 80 | 525.38±78.95 | 1340.17±36.15 | 303.04±16.31 | 341.61±63.38 | 4462.26±1332.42 |
| | C263F | 64 | 226.58±35.39 | 604.52±4.26 | 188.86±15.86 | 353.30±9.88 | 2975.94±159.60 |
| | E273A | 63 | 254.63±11.88 | 1102.78±52.85 | 251.01±23.33 | 559.75±128.60 | 3314.43±711.69 |
| | A79V | 62 | 381.33±25.78 | 883.01±75.02 | 245.54±12.29 | 475.93±43.49 | 5450.24±151.04 |
| | E123K | 62 | 513.07±93.97 | 1347.99±54.41 | 297.93±10.50 | 365.10±68.78 | 4375.57±1659.96 |
| | A409T | 58 | 368.26±26.09 | 1103.78±73.15 | 287.94±25.21 | 470.76±120.36 | 4266.68±650.79 |
| | E280A | 47.4 | 77.92±9.13 | 297.73±9.34 | 127.37±1.64 | 454.38±10.10 | 2046.95±257.45 |
| | M233T | 46 | 398.91±41.74 | 398.50±38.73 | 491.73±18.85 | 186.55±16.18 | 5081.20±188.82 |
| | M146L | 45 | 339.95±65.62 | 857.45±88.55 | 307.88±6.05 | 200.24±19.98 | 4272.82±748.31 |
| | G384A | 37 | 133.13±15.80 | 176.32±20.69 | 169.06±8.98 | 240.78±51.25 | 1838.91±102.32 |
| | I143T | 34 | 151.32±33.79 | 453.35±30.43 | 320.20±21.04 | 262.09±11.12 | 3383.50±194.50 |
| | L166P | 24 | 63.55±7.24 | 265.53±22.90 | 67.89±8.04 | 612.18±147.29 | 1930.73±390.55 |
| HEK | WT | 80 | 520.64±44.87 | 1156.44±82.00 | 248.34±9.91 | 262.17±19.93 | 4237.70±943.51 |
| | H163R | 68 | 396.45±21.39 | 1194.98±88.06 | 441.37±16.38 | 412.48±48.86 | 5132.14±755.48 |
| | delE9 | 50 | 63.55±11.71 | 310.78±31.60 | 37.28±21.93 | 308.46±10.91 | 1934.57±722.97 |
| | I213T | 48 | 163.59±8.67 | 731.04±24.28 | 293.15±8.65 | 393.69±31.69 | 3915.83±49.00 |
| | L286V | 47.5 | 537.50±37.14 | 1264.73±94.91 | 316.44±5.58 | 331.12±27.74 | 4915.31±414.63 |
| | M233L | 46 | 357.26±10.44 | 984.11±99.20 | 472.27±20.30 | 303.19±6.43 | 4430.39±1777.08 |
| | G206V | 37 | 202.39±45.20 | 824.37±52.44 | 721.92±88.59 | 228.34±6.29 | 7256.93±1383.59 |
| | L381V | 29 | 250.23±34.98 | 1058.47±109.79 | 466.58±5.87 | 1584.64±28.76 | 10113.72±671.58 |
| | L85P | 27 | 66.38±5.25 | 160.97±9.14 | 75.27±8.24 | 365.34±12.97 | 1383.56±210.18 |
