# Supplementary figures and images for: Switched Aβ43 generation in familial Alzheimer’s disease with presenilin 1 mutation
Source: Transl Psychiatry. 2021 Nov 3;11:558. doi: 10.1038/s41398-021-01684-1 (PMC8564532; doi:10.1038/s41398-021-01684-1)

Fig. S1

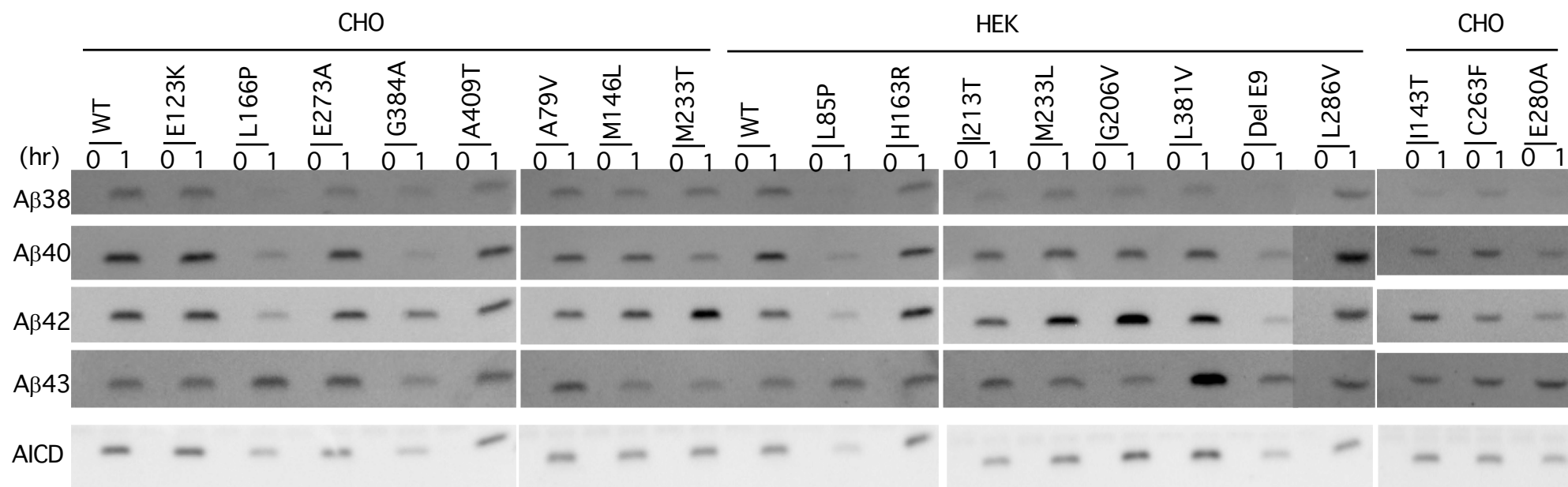

Supplement: Supplementary file 2 — Fig. S1 [file 41398_2021_1684_MOESM2_ESM.pdf]

Fig. S2

A

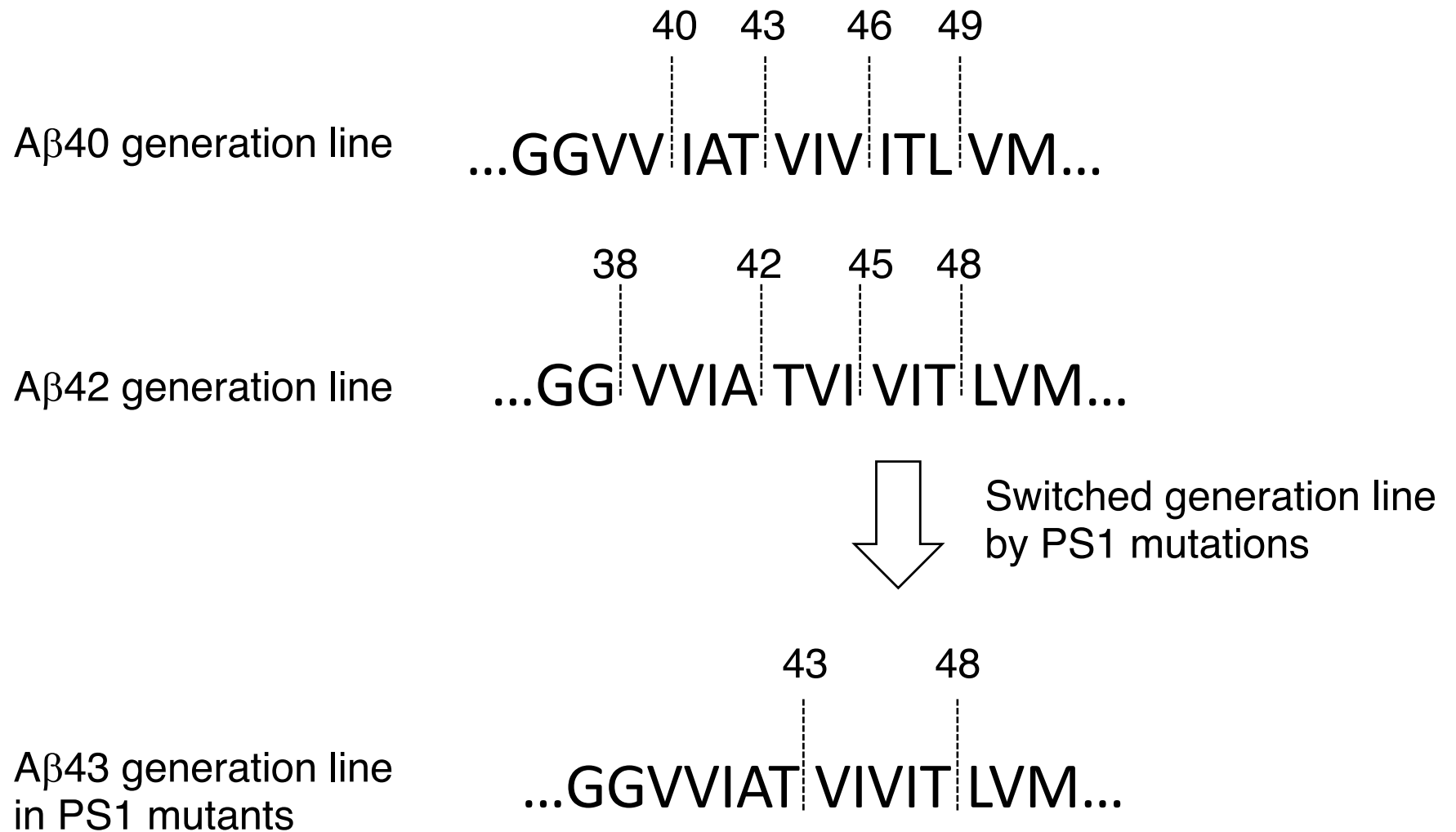

Fig. S2

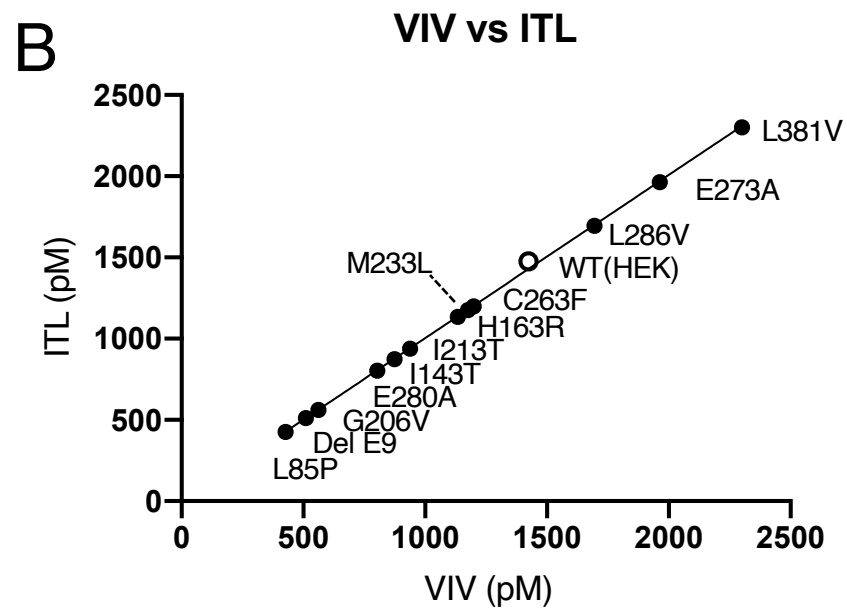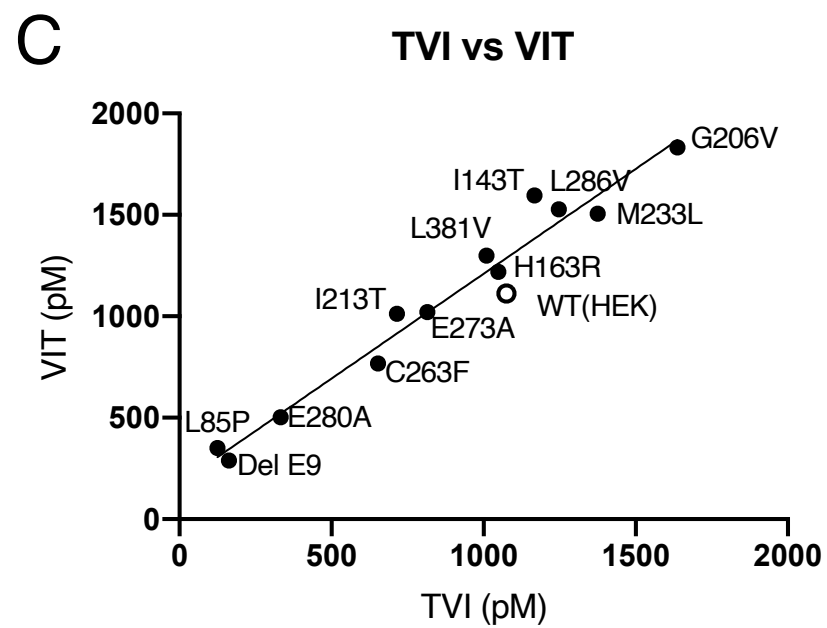

Supplement: Supplementary file 3 — Fig. S2 [file 41398_2021_1684_MOESM3_ESM.pdf]

Fig. S3

A

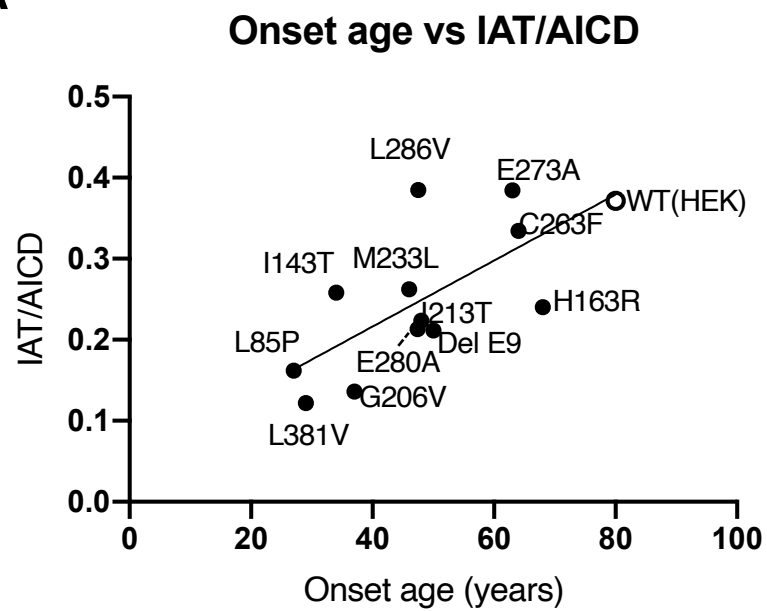

B

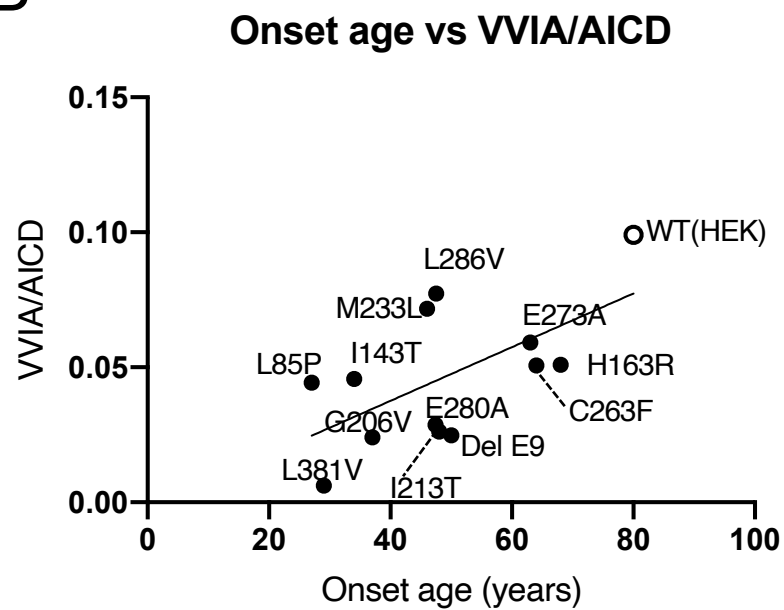

Supplement: Supplementary file 4 — Fig. S3 [file 41398_2021_1684_MOESM4_ESM.pdf]

Fig. S4

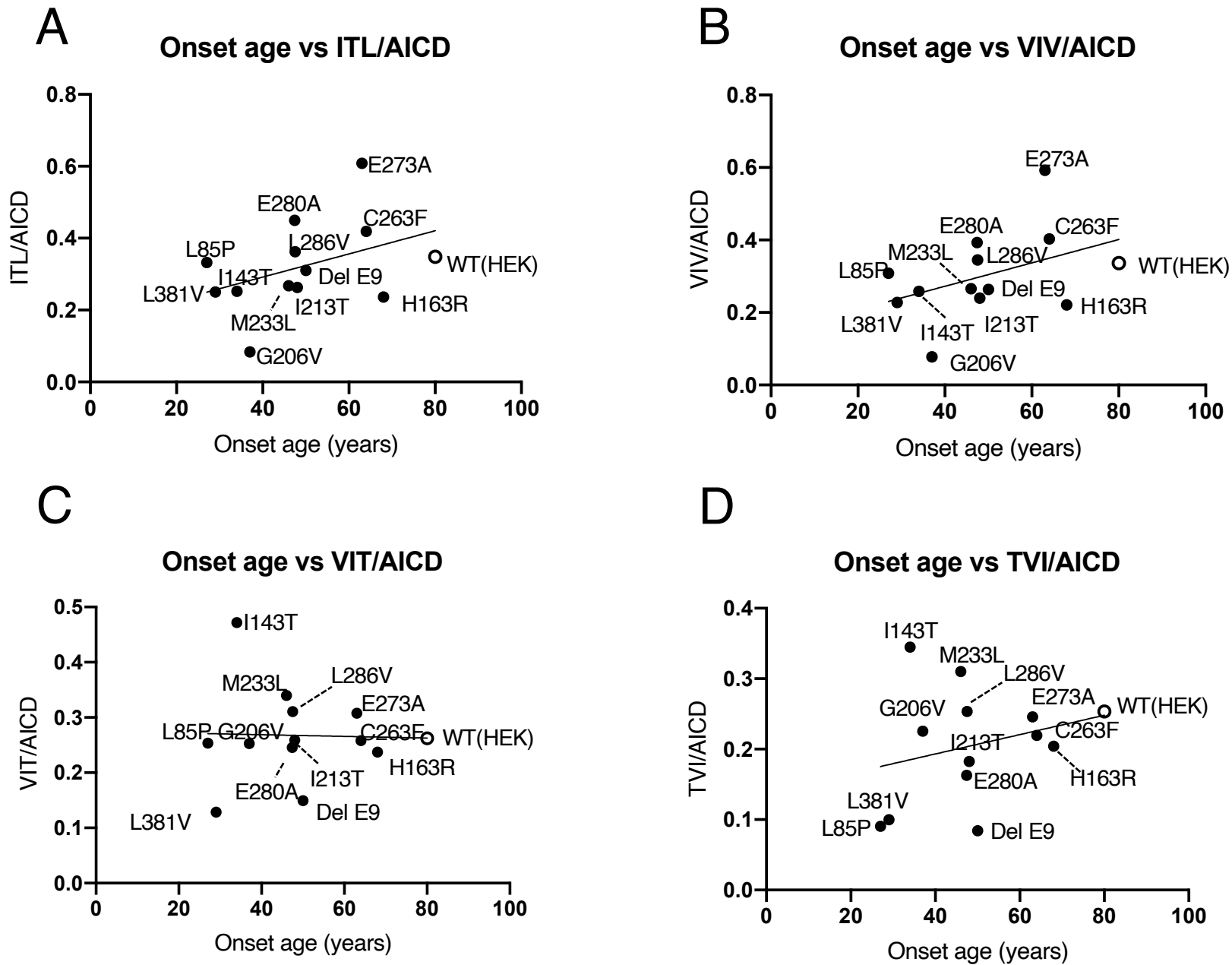

Supplement: Supplementary file 5 — Fig. S4 [file 41398_2021_1684_MOESM5_ESM.pdf]
