# Supplementary material for: Switched Aβ43 generation in familial Alzheimer’s disease with presenilin 1 mutation
Source: Transl Psychiatry. 2021 Nov 3;11:558. doi: 10.1038/s41398-021-01684-1 (PMC8564532; doi:10.1038/s41398-021-01684-1)
Supplement: Supplementary file 6 — Supplementary legends [file 41398_2021_1684_MOESM6_ESM.docx]

**Supplementary information**

**Table S1**

Each generated Aβ and AICD was transformed to pM after calculations with authentic Aβ and AICD standard. (n=3)

**Supplementary Figure S1**

**The generated Aβ38, Aβ40, Aβ42, Aβ43, and AICD γ-secretase assays were performed *in vitro*.** WT or FAD mutant PS1 containing lipid raft-associated γ-secretase fractions incubated with 500 nM C99-FLAG for 1 hr. Each generated Aβ and AICD was detected with specific antibodies. (n=3).

**Supplementary Figure S2**

**The sequencing released peptides by stepwise processing.** (A) The position of cleaved and released peptides in the stepwise processing. (B) ITL was released from Aβ49 to Aβ46, and VIV was released from Aβ46 to Aβ43. (C) VIT was released from Aβ48 to Aβ45, and TVI was released from Aβ45 to Aβ42. The open circle indicates WT PS1 and the closed circles indicate mutant PS1. (n=3).

**Supplementary Figure S3**

**FAD onset age versus the ratios of IAT/AICD or VVIA/AICD.** (A) IAT was generated from Aβ43 to Aβ40. (B) The VVIA was generated from Aβ42 to Aβ38. These relationships indicated that Aβ38 and Aβ40 generation decreased from those of precursors, Aβ2 and Aβ43, in onset age dependency. (n=3).

**Supplementary Figure S4**

**Onset age versus the ratios of released peptide/AICD.** (A) ITL/AICD; ITL/AICD=0.003222 x (onset age) + 0.1629, R=0.1639, (B) VIV/AICD; VIV/AICD=0.003226 x (onset age) + 0.1433, R=0.1773, (C) VIT/AICD; VIT/AICD= -0.0001513 x (onset age) + 0.2749, R=0.0008049, and (D) TVI/AICD; TVI/AICD=0.001373 x (onset age) + 0.1383, R=0.07211. (n=3).
